# Treatment planning for patients with acoustic neuroma

**DOI:** 10.3389/fonc.2025.1645881

**Published:** 2025-11-06

**Authors:** Andrew J. Fishman, Oskar Rosiak, Arnaldo Rivera, Michael Puricelli, Nenad Zivkovic, Jozef Mierzwinski, Bojan Markovic, Natalija Milisavlevic, Radosław Rola, Marcin Szymanski

**Affiliations:** 1 Department of Otolaryngology–Head & Neck Surgery, University of Missouri, Columbia, MO, United States; 2 Department of Otolaryngology–Head and Neck Surgery, Medical University of Lublin, Lublin, Poland; 3 Department of Otolaryngology, Audiology and Phoniatrics, Children’s Hospital of Bydgoszcz, Bydgoszcz, Poland; 4 Department of Neurosurgery and Pediatric Neurosurgery, Medical University of Lublin, Lublin, Poland; 5 Department of Otolaryngology, Polish Mother’s Memorial Hospital Research Institute, Lodz, Poland; 6 Department of Pediatric Otolaryngology–Head and Neck Surgery, Children’s Mercy Kansas City, Kansas, MO, United States; 7 Department of Neurosurgery, Euromedic Hospital, Belgrade, Serbia; 8 Department of Otolaryngology, BelMedic Hospital, Belgrade, Serbia; 9 Department of Otorhinolaryngology, University Clinical Center Niš, University of Niš, Niš, Serbia

**Keywords:** acoustic neuroma, schwannoma, gamma knife, stereotactic radiation, middlecranial fossa, suboccipital approach, translabyrinthine approach, schwanomma surgery

## Abstract

**Introduction:**

Acoustic neuroma (AN) is a benign tumor of the vestibulocochlear nerve, with increasing detection due to improved imaging. Treatment decisions are complex, requiring an individualized approach based on tumor size, location, growth rate, and patient-specific factors such as hearing and vestibular function.

**Results:**

Treatment options include observation, microsurgery, and stereotactic radiosurgery. Hearing preservation is prioritized in select cases using middle cranial fossa or retrosigmoid approaches, while translabyrinthine surgery is preferred for larger tumors or disabling dizziness. Stereotactic radiosurgery offers a non-invasive alternative but has variable long-term hearing outcomes and potential tumor regrowth. Vestibular rehabilitation is essential post-treatment, particularly after surgical resection.

**Discussion:**

Decision-making depends on patient age, tumor progression, and symptom severity. Younger patients with small tumors may benefit from early intervention to preserve hearing, while older patients with severe imbalance achieve better quality-of-life outcomes with surgery. Stereotactic radiosurgery remains an option for patients unable to undergo surgery, though its long-term efficacy and side effects require careful consideration. A multidisciplinary approach is essential to optimize treatment outcomes.

**Summary:**

AN management must be tailored to individual patient profiles. This review integrates current literature and expert clinical experience to guide otolaryngologists, neurologists, and oncologists in treatment planning. Future research should refine treatment algorithms and improve functional outcomes.

## 1 Introduction

The reported annual incidence of sudden sensorineural hearing loss (SSNHL) in the 21st century is 27 per 100, 000 (1). Acoustic neuroma (AN) is identified as the cause in 5% of cases when an MRI is performed (2). The most cited historical epidemiological data for the overall prevalence of acoustic neuroma, is one per 100, 000 persons (3), which seems rather low given the SSNHL data. Furthermore, only 10% of patients with known acoustic neuromas experience sudden hearing loss, and 7.7% have experienced two or more episodes of sudden hearing loss (4). So, it quickly becomes apparent that the real prevalence of acoustic neuroma may be much higher.

Studies conducted in Europe reveal an increase in the European Standardized Rate (ESR) of AN from 10.3 to 15.5 per million inhabitants; however, the authors highlighted regional variations, with incidence rates ranging from 12.0 to 24.9 per million, which is likely due to the availability of diagnostics and data collection (5). In another study from the US, Robert Jackler from the University of California reviewed a database of over 45, 000 MRIs and found the prevalence of undiagnosed acoustic neuroma may be 2 out of every 10, 000 persons (6). In a study from Denmark spanning over 40 years of long-term observation, an average annual incidence rate of 19.42 per million person-years was reported. The authors noted that the observed increase might be due to heightened awareness and advancements in imaging technologies (7). As MRI has become widely available and affordable the detection of acoustic neuroma is likely to increase in Europe and worldwide.

Treatment planning for acoustic neuroma must be based upon each patient’s specific clinical presentation. The major factors to be considered are hearing and dizziness, tumor size, location & rate of growth, patient age, medical, surgical and radiation risk, and finally, patient choice.

The decision should also consider the experience and skills of the treating team. Data from the specialists, as well as data from the literature, must be considered carefully and guide the decision. There needs to be a frank discussion about the predicted outcomes of all treatment options in both the long and short terms. If any additional imaging, physiologic testing, or medical evaluations are requested, then the options should be reexplored. Ultimately, the decision will come down to three options: observation with imaging over time; removal by microsurgery; or radiation therapy. What may be an ideal solution for one patient may, however, be a bad option for another.

Currently, several treatment options are available for patients with AN. However, there is no single comprehensive guideline regarding treatment decisions for AN patients. There are some official systematic reviews and meta-analyses from major neurosurgical, otolaryngological and oncological associations. A systematic review from the Congress of Neurological Surgeons from 2018 recommends observation over radiosurgery for small tumors when growth is not documented (8). By contrast, a recent V-REX clinical trial published in 2023 recommends upfront radiosurgery for small- to medium-sized tumors rather than observation. However, it notes that hearing preservation rates at the 5-year mark are similar (9). A recent meta-analysis of long-term hearing outcomes for vestibular schwannomas after microsurgery and radiotherapy revealed a much higher prevalence of serviceable hearing following microsurgery (a pooled estimate of 74.5%) than after radiosurgery (18.1%) at 10-year follow-up, and clearly showed a decline of serviceable hearing at the 5-, 7- and 10-year marks (10).

The aim of this narrative review is to present the treatment options for AN patients, taking into consideration specific clinical presentation and patient factors and present these ideas based on clinical cases from the Authors’ practice. The senior author, AF, has consulted and surgically treated over 500 cerebellopontine angle (CPA) lesions in Europe, Asia and North America, over nearly 30 years. The senior authors are also trained and certified in the use of stereotactic radiation techniques. The opinions expressed are a culmination of these clinical experiences and a careful review of the international literature.

## 2 Symptoms and presentation, patient-dependent factors

### 2.1 Hearing

We must consider the present level of hearing, sudden recent changes, and the hearing on the other side. This should be compared to the expected hearing if the tumor is left untreated, after radiation, and from different microsurgical methods. Both short- and long-term expectations should be considered. Some patients present with the rare disease of Type 2 Neurofibromatosis (NF2) and bilateral tumors, complicating the treatment process. In these patients, the long-term natural history may include deafness in both ears and other neurological issues unique to the disease. Fortunately, 95% of patients with acoustic neuroma do not have NF2 and present with a single-sided tumor.

### 2.2 Vestibular symptoms

Several studies have examined the prevalence and onset of vestibular symptoms in patients with acoustic neuroma. While the most common first symptom of AN is hearing loss, vertigo attacks are present in 12% of patients as first symptoms, but as many as 61% of patients experience dizziness during the disease (11). These symptoms often appear once treatment commences and are often neglected by managing physicians. The effect of vestibular symptoms on quality of life depends on the severity, duration, timeframe, and other comorbidities that may impact vision and balance. If vestibular symptoms do develop, they can often lead to a significant reduction in quality of life, an increase in risk of falls, and account for increased comorbidity in the elderly. If dizziness and balance are a primary concern, it is often best treated surgically (12). Vestibular rehabilitation should be administered to every patient suffering from vertigo or dizziness with AN, especially when symptoms develop due to unilateral vestibular hypofunction after surgical treatment (13).

### 2.3 Size and location

**Figure 1:**
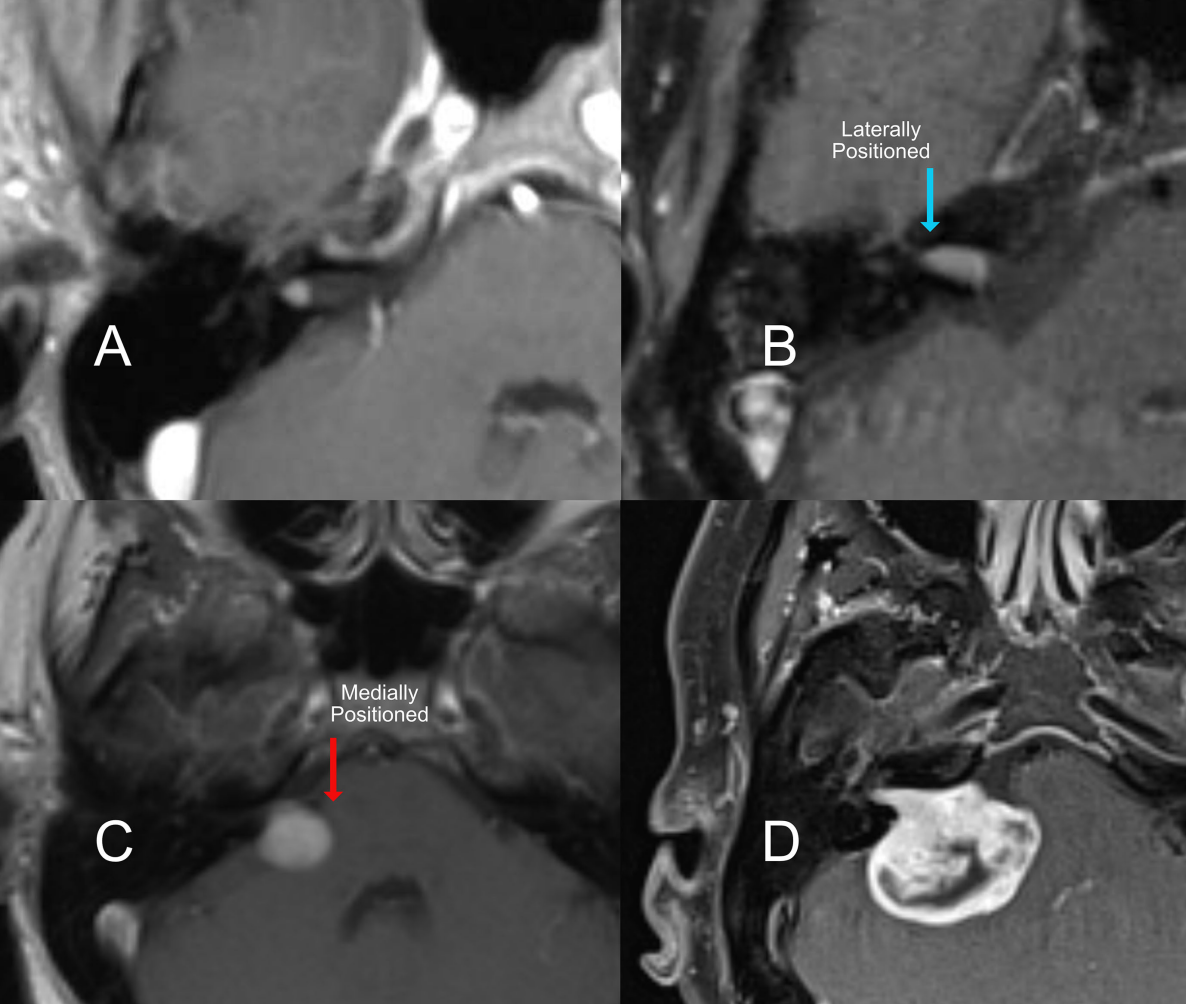
T1 Contrast MRI of an acoustic neuroma with different locations relative to internal auditory canal. **(A)** a very small intracanalicular tumor, **(B)** a laterally positioned tumor filling the auditory canal, **(C)** medium tumor medially positioned to the internal acoustic canal, and a large tumor **(D)** with brainstem compression.

The internal auditory canal is the most common site of origin for these tumors. It is about 1 cm in length, which serves as a visual reference for size estimation. Modern software systems precisely measure tumors in 3 dimensions, which is especially helpful when observing a tumor over time. Though tumor size is reported and discussed as linear measurements, the volume of the tumor is a more accurate representation of its size. Approximating the tumor as a sphere, the volume would be 4/3 π r^3^. Doubling the tumor radius would yield an 8-fold increase in volume **Figure 1**.

The location of the tumor relative to the medial and lateral ends of the internal canal is also important. Tumors that do not go all the way to the lateral end of the internal canal are less damaging to the gentle blood supply and fine nerves entering the organ of hearing. As a tumor grows to fill this space, the likelihood of preserving hearing with microsurgery decreases (**Figure 1B** & C). However, if tumors grow untreated (**Figure 1D**), it is also more likely that the hearing will decline on its own over time. Location also affects the hearing outcome from radiation. The closer the tumor is to the cochlea (**Figure 1A**), the higher the collateral dose of radiation delivered to the hearing organ, which will impact the long-term hearing outcome.

### 2.4 Rate of growth

Acoustic neuromas, in general, are considered mostly benign and slow-growing. However, the rate of growth varies between patients. Research into the biology of schwannoma tumors has identified several molecular pathways that may influence growth rates and account for this individuality. One study highlighted the role of the phosphoinositide 3-kinase (PI3K) and focal adhesion kinase (FAK) pathways in schwannoma cell proliferation (14). The researchers found that inhibiting these pathways reduced tumor cell growth, suggesting their involvement in tumor progression. Additionally, the merlin protein, encoded by the NF2 gene, is a known tumor suppressor whose loss is associated with schwannoma development. Variations in the degree of merlin dysfunction may contribute to differences in tumor growth rates among patients; however, why the growth rate can change dramatically in the same patient remains unknown and requires further research.

Studies on growth rate typically consider slow growth to be 0.3 mm or less per year and fast growth to be 4 mm or more (15). However, an acceleration of growth may occur, and studies found that 33% of newly diagnosed tumors grow in the follow-up period of 1–3 years; however, by 5 years, this increases to 50% (16).

### 2.5 The factor of age

The treatment plan is often affected by the patient’s age. However, advanced age does not always correlate with increased risk. A study on the incidence of AN in England found that the incidence rate peaks in the 60–69 age group to the range, at 5.7–6.1 per 100, 000 person-years and that age was negatively correlated with tumor size. This is likely due to an increase in the diagnosis of small tumors with a long duration of audio-vestibular symptoms in older patients compared to earlier studies (17). While stereotactic gamma radiation treatment (commonly known as “gamma knife”) may seem a straightforward option for older patients, it is often feasible to simply monitor the tumors with periodic MRI scans. Surgery in the older patient is also a reasonable option if their symptoms would directly benefit from it and the patient is healthy enough, as some elderly patients present with severe balance symptoms that are best resolved with surgery and vestibular rehabilitation. We stress this point because it is important not to dismiss patients for surgery based only on age, thereby depriving them of treatment that could improve their quality of life. Bruce Gantz from the University of Iowa reported that surgery is quite safe and appropriate in patients aged 65 and older when appropriately indicated and the patient is healthy (15).

In children, acoustic neuromas have been reported as young as 4 years old (18, 19). Individuals diagnosed prior to 25 years old are more likely to have a tumor predisposition syndrome and are recommended to undergo evaluation, including genetic testing for such conditions (20). The most frequent predisposing condition is NF2, for which modified diagnostic criteria and nomenclature were agreed upon by the International Consensus Group on Neurofibromatosis Diagnostic Criteria in 2022 (21). Tumors in pediatric patients are more proliferative, and symptoms from mass effect are more common. While treatment options are the same as in adults, residual pediatric tumors have a higher regrowth rate (22).

## 3 Surgical treatment

**Figure 2:**
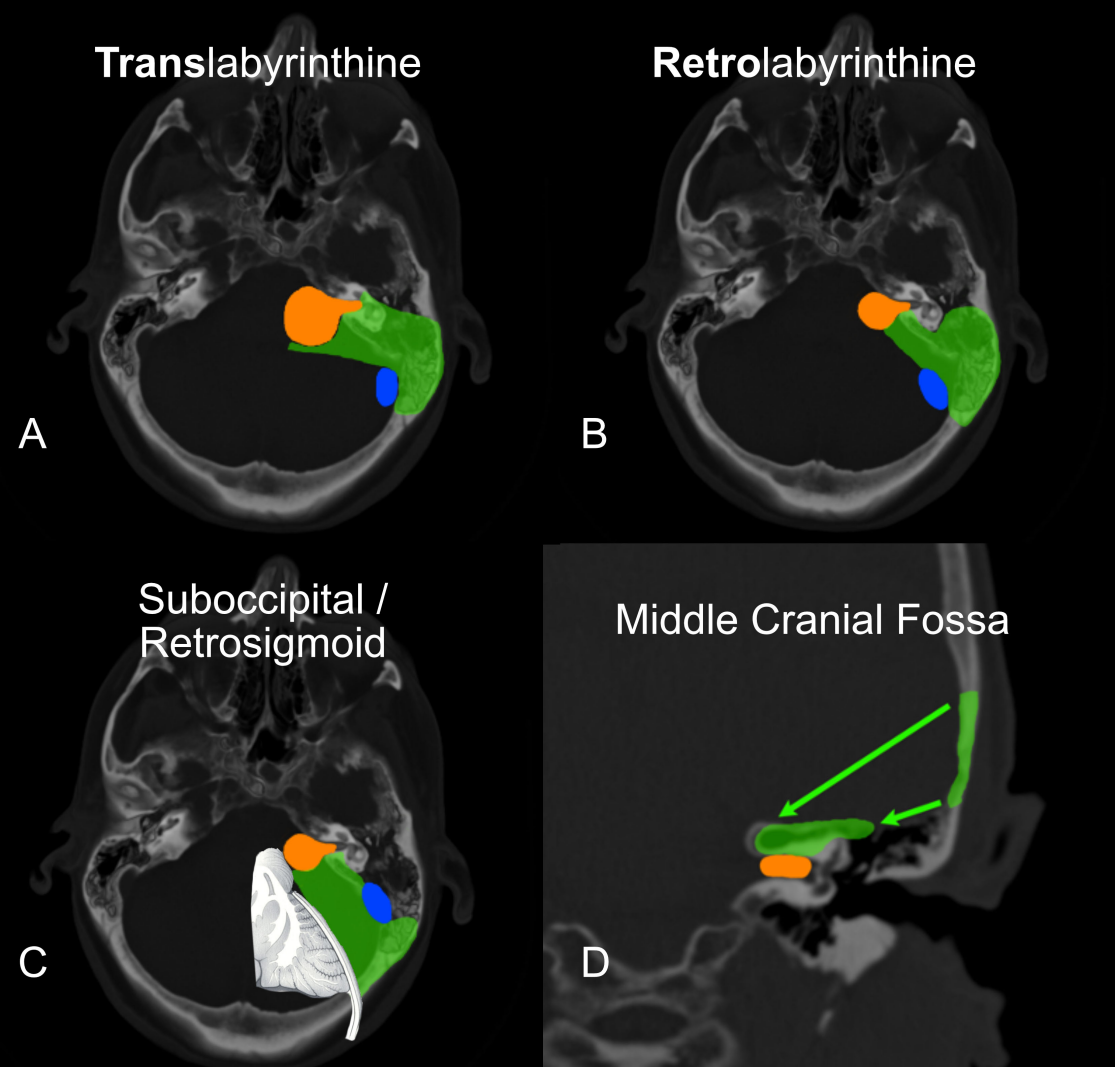
The transtemporal approaches are diagrammed in: **(A)** Translabyrinthine & **(B)** Retrolabyrinthine hearing preservation variants. The Suboccipital/Retrosigmoid approach is seen in **(C)** (Note the significant brain retraction required with this approach). The middle cranial fossa is diagrammed in **(D)**. In all panels, the green delineates the bone removed and the view to the tumor. The tumor is in orange. The sigmoid sinus is in blue.

There are two broad categories of approaches: those that aim to preserve remaining hearing (**Figure 2B, C, D**) and those that do not (**Figure 2A**). The selection of approach is dependent on the goals of the operation and the clinical presentation.

**Figure 2**.

The middle cranial fossa (MCF) approach is used when the tumor is small and isolated to the internal auditory canal. (**Figure 2D**) It is designed to preserve the organ of hearing and its function. The probability of hearing preservation as reported from experienced centers is around 70% (23, 24). This is clearly the case in our experience as well. The best chance is when the tumor is smallest. Accordingly, it is indicated to operate electively in young patients with the smallest of tumors. More importantly, the likelihood of retaining this hearing level for the long term is as high as 98% when looking at speech discrimination and 82% when looking at pure tones alone (23). So, the preserved hearing is durable over time when the tumor is removed.

This operation requires that a small square of bone be removed ***above*** the inner ear structures. The temporal lobe of the brain is kept covered and protected by the fibrous dura mater and elevated just enough to safely do the surgery. Removal of 20–30 ml of spinal fluid either from the lumbar spinal area or directly from a small incision in the temporal lobe dura reduces the intracranial pressure. The lower pressure makes the elevation of the temporal lobe safer. MCF is limited in that it cannot safely reach medially towards the brainstem and is reserved for smaller tumor sizes in which the chances of hearing preservation are highest.

The retrosigmoid approach, also called the suboccipital approach (as it is performed below the occipital cortex of the brain), is one of the first approaches developed for the removal of acoustic neuroma. It also requires craniotomy (**Figure 2C**). It has the potential benefit of providing a large opening and can be used in many cases of varying tumor sizes. It also has the benefit of potentially preserving the inner ear and hearing. It does, however, require direct compression of the brain without protection from the dura. This approach has been used for decades, and many patients can still benefit, especially in cases of meningioma that spread along the dura and extend back under the sigmoid sinus.

The remaining approaches are called transtemporal because they go directly through the mastoid and temporal bone. They offer the distinct advantage of avoiding brain compression, as the tumor is approached directly through the inner ear and mastoid. The translabyrinthine approach is the foundation of these approaches(**Figure 2A**). The mastoid portion of the temporal bone is opened immediately behind the ear. The balance canals of the inner ear are removed, and the tumor is removed through the temporal bone, avoiding brain retraction. The retrolabyrinthine approach is a modification of the translabyrinthine approach and offers the possibility of hearing preservation (**Figure 2B**). This modification is limited to smaller tumors that do not grow out into the lateral internal auditory canal. The translabyrinthine approach has also been modified and described for use in very large tumors as the “expanded transtemporal approach” (25).

The translabyrinthine approach does not aim to preserve residual hearing. It does, however, have the lowest rate of complications of spinal fluid leak and has a recurrence rate of 1% (26, 27). The transtemporal approaches permit safe tumor removal, regardless of size. They provide early identification of the facial nerve in the ear and mastoid, as well as in the deeper brainstem region where it originates. Personal series show good facial nerve function after surgery in 90% of the largest tumors (3 cm or greater) and even better for smaller ones (28). The resulting sudden unilateral vestibular loss generated by cessation of vestibular input from one of the labyrinths may be a debilitating symptom. However, due to the natural compensation mechanisms, these symptoms resolve in three months (29). Translabyrinthine tumor removal is the best treatment for dizziness and imbalance, especially when compared to radiation and continued observation (12, 30).

While it has often been argued that the retrosigmoid approach (**Figure 2C**) offers a chance at hearing preservation in all cases, the likelihood is quite low. When the tumor is 2 cm or larger, the chance of hearing preservation is less than 5% (31–34). The likelihood for tumors between 1.5 and 2.0 cm is around 17% via the retrosigmoid approach. Noel Cohen from New York University (NYU) found that no tumors 2.5 cm or larger had successful hearing preservation via the retrosigmoid approach (33). Meningiomas, on the other hand, tend to grow one anatomical layer away from the nerves, so it is still a useful approach in some patients with this pathology. The recurrence rate after retrosigmoid surgery is 10%, which is 10 times higher than tumors operated on translabyrinthine. The earliest publication from NYU demonstrated a 21% CSF leak rate (26). The technique was then modified reducing the rate to 10% (27). This is still twice the rate compared to the translabyrinthine approach. The transtemporal approaches also have the major advantage of reducing postoperative brain swelling by avoiding brain retraction. Consequently, they tend towards a much faster postoperative recovery. Given the unlikelihood of hearing preservation with medium and larger size tumors, our team prefers the safety of transtemporal surgery. The translabyrinthine approach is the preferred treatment for patients with vertigo, patients with poor hearing, and patients with tumors too large for hearing preservation.

For patients with small to medium vestibular schwannomas, particularly in cases of NF2, complete tumor resection followed by cochlear implantation (CI) represents an ideal therapeutic approach (35). This strategy offers the potential for meaningful auditory rehabilitation while addressing the tumor definitively (36). In contrast, the long-term tumor control rates associated with low-dose radiation remain uncertain, and the risk of progressive hearing loss persists, even with initial preservation attempts. Therefore, early surgical intervention combined with CI should be considered a favorable treatment paradigm, especially in patients for whom hearing function is a priority.

## 4 Stereotactic radiation

Several systems are available to treat both malignant and benign tumors using radiation delivered by 3D stereotactic plans. The most widely used system in the US and Europe is the Leksell Gamma Knife^®^, which uses 192 stationary sources of radioactive *cobalt-60*. Alternatively, the CyberKnife^®^ uses linear acceleration (LINAC) from a moving source to deliver high-energy radiation from a computer-controlled robotic arm. During Gamma Knife^®^ radiation, the patient’s head is held in place by a metal frame with four pins. The patient is first placed in an MRI, which produces a 3D map of the patient and tumor for planning the radiation treatment. With the frame still attached, the patient is then moved onto a sled and exposed to multiple small doses of radiation from different angles. These doses overlap to deliver the highest dose to the tumor. These individually programmed exposures, called “shots” in the Gamma Knife^®^ plan, correspond to computer-controlled openings of the doors blocking the radioactive cobalt or to movements of the arm in LINAC-based systems. Though some systems use a custom-made synthetic mask placed over the eyes and face to hold the head in place, the greatest accuracy is achieved by using the frame directly attached to the skull with metal pins to suppress movement. The exposure usually lasts around 30–90 minutes, depending on the dimensions of the tumor. Patients are not put under anesthesia, but sometimes require some intravenous sedation to remain still.

Another treatment option that has been explored for vestibular schwannoma is proton beam therapy, which uses charged particles to deliver a conformal radiation dose while aiming to minimize exposure to surrounding tissues. However, current evidence does not demonstrate clinical superiority over Gamma Knife^®^ radiation.

A large cohort study by Koetsier et al. (37) reported a median follow-up of 4.5 years (54 months), while Vernimmen et al. (38) observed a mean clinical follow-up of 6 years. However, both intervals remain insufficient to confirm durable long-term control or assess late toxicity outcomes.

In a recent systematic review, Santacroce et al. (39) found that tumor control and cranial nerve preservation with proton therapy were comparable to Gamma Knife^®^ radiation, but hearing preservation rates were generally lower. Furthermore, proton therapy remains substantially more costly than Gamma Knife^®^ radiation without clear evidence of added clinical benefit.

It is noteworthy that the first author of the present review was affiliated with one of the few proton centers in the United States, at Northwestern Medicine^®^, where the clinical team did not treat acoustic neuroma patients with proton beam radiation.

### 4.1 Dizziness and radiation

Andrew Parsa from the University of California San Francisco, USA, noted that dizziness in patients with acoustic neuroma is a “relatively understudied complaint … despite its significant impact on quality of life … highlighting the importance of this symptom” (40). Magid Samii from the University of Hannover supports this view, as reported in the Journal of Neurosurgery in 2017. He stated: “Disabling vestibular dysfunction that affects quality of life should be considered an indication for surgery, even in otherwise asymptomatic patients with intracanalicular acoustic neuromas. Surgical removal of the tumor is safe and very effective in regard to symptom relief” (41). Bojrab et al. from the Michigan Ear Institute, USA recently published results on 98 patients treated with stereotactic radiation and found that 47% experienced acute vestibulopathy. They also reported that “vestibular symptoms were reported at a significantly higher frequency among subjects who had reported vestibular symptoms before their treatment (p=0.001)” (42). Lee et al., at Mount Sinai Medical Center in New York, USA, reported in a study of over 100 patients that about one-third of patients developed new vertigo after radiation (30). Approximately 20 years earlier, in 2006, Hempel et al. from Munich followed 123 patients for a mean time of 8 years after radiation and observed that “13% experienced vertigo for the first time.” They also found that, “6% reported [new] trigeminal neuralgia.” They reported the hearing preservation rate to be “equivalent to microsurgery” (43).

### 4.2 Radiation and hearing preservation

While much has been reported about the hearing outcomes after stereotactic radiation, data following patients for 10 years or longer are few. The Neurosurgery Department at the University of Pittsburgh, USA, is tasked with training most American neurological surgeons in Leksell Gamma Knife^®^ radiation treatment. In 2015, the group presented available outcomes for patients who underwent radiation at least 10 years prior. Only 12% had good hearing, another 12% had serviceable hearing (adequate for hearing aid use) by the Gardner–Robertson scale, and 76% had either no hearing or hearing too poor to benefit from hearing aids. Tinnitus and balance were not discussed. The Gardner–Roberson scale is accepted by the AAO-HNS to assess hearing outcomes after acoustic neuroma treatment (44). A recent meta-analysis pooling the results of 9 studies with 965 patients revealed that the long-term outcomes in terms of serviceable hearing after radiosurgery gradually worsened, reaching 18.1% at the 10 year follow-up. The authors conclude that long-term hearing outcomes with microsurgery remain much higher at 74.5% and stable over time, with the highest prevalence of hearing loss in the post-surgical period, which is understandable, since different surgical techniques were pooled. Golfinos et al. (45) also noted that radiosurgery was superior in hearing preservation due to shorter patient follow-up periods.

## 5 Discussion

### 5.1 Case example of leksell gamma knife^®^ treatment

**Figure 3:**
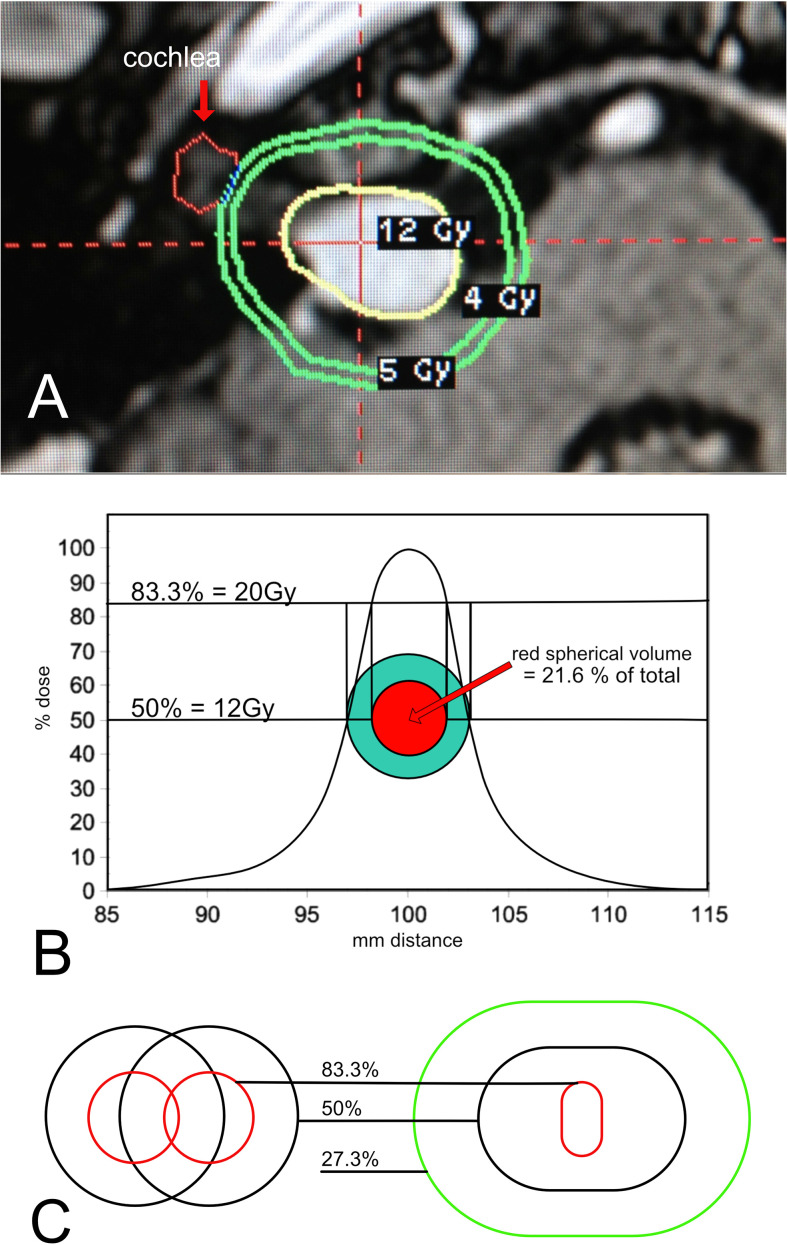
**(A)** Gamma Knife^®^ plan for a medium sized tumor deemed appropriate for treatment. **(B)** Single Shot Dose Curve. A single shot reference isodose curve from a 4mm collimator. % isodose is represented on the y-axis. The x-axis is mm from the center. Note that the curve generates an equivalent circular trace in green that has an edge trace of 12Gy. As the curve moves towards the maximum, the red trace generated by the 83.3% dose correlates to the dose of 20Gy. 100% will fall at the center point and have a 24Gy maximum dose. The volume of the red sphere is only 21.6 percent of the total green sphere volume. **(C)** Normalization of two shots. Two individual shots are displayed on the left. The radiation physics of Gamma Knife^®^ dictates that when these shots are combined as in actual plans, the higher dose lines compress, as they are seen in the figure on the right. The resulting dose covers a much smaller volume at the 83.3 percent isodose line.

An 82-year-old patient demonstrated active growth in a previously stable tumor. There were no symptoms of dizziness or imbalance, but hearing impairment was mild. The patient had a history of cardiovascular disease. The tumor had doubled in length over the past 3 years, reaching a size of around 1.5 cm in maximum measurement. The tumor was positioned more medially, with most of the tumor volume outside the internal auditory canal. (**Figure 3A**). The patient was indicated for treatment, but, due to age and increased surgical risk, lack of vestibular symptoms, and good hearing status, a decision was made by the team and the patient to use Leksell Gamma Knife^®^.

**Figure 3**.

Like treatment planning for surgical candidates, age, dizziness, hearing, and the size and position of the tumor are considered when treating with stereotactic radiation. By comparison, if the patient were younger and in good health, retrolabyrinthine hearing preservation microsurgery would be offered. For a patient of the same age but with better cardiovascular condition who was primarily bothered by imbalance and dizziness, surgical removal by either the translabyrinthine or retrolabyrinthine approach would be appropriate. The translabyrinthine approach does not preserve hearing but is the most effective treatment for vestibular complaints. Godefroy et al. support this view, concluding in a prospective study that patients with disabling vertigo experience significantly reduced quality of life. Consequently, translabyrinthine tumor removal should be considered in patients not only with larger tumors but also with small- and medium-sized tumors (12). Surgery is preferred with larger tumors, where the larger spread of radiation to the brainstem would be associated with unwanted damage. This opinion is strongly supported by Møller, who expressly stated that “large tumors should be operated, as should most medium-sized, as should smaller tumors with persistent symptoms of vertigo…” Residual and recurrent tumors after surgery can be considered for Gamma Knife^®^ (46). When considering all the side effects of radiation, Kim et al., from Seoul, South Korea, reported in 2017 in World Neurosurgery an update on 235 patients with a minimum of one year follow-up. They found “…diminished facial motor function in 6%, facial spasm in 11%, hydrocephalus in 6.4% and trigeminal neuralgia in 9.4%…” of patients treated with stereotactic radiation. They also found that patients receiving “…13Gy or more of radiation at the tumor margin had a significantly higher probability of loss of serviceable hearing … [and that] older patients had a significantly higher probability of vestibular nerve dysfunction” (47).

In our experience, translabyrinthine microsurgery is preferred in older patients because the surgery is safer and associated with excellent outcomes and low complication rates. This sentiment is echoed by Gantz et al., who reported on 100 patients aged 65 and over with tumors less than 2.5 cm. They concluded that “management should be based on biological age, “ and that surgical treatment can safely be performed (15). They demonstrated that up to one-third of older patients may express growth rates averaging 4mm or more per year, requiring surgery, while two-thirds could be observed if the growth rate were 0.3 mm or less.

### 5.2 Radiobiology of gamma knife^®^ and changing trends in treatment dose

Gamma Knife^®^ is administered in a series of “shots.” The shots are layered upon one another to create the final shape of the radiation dose. In **Figure 3A**, we see that the shape is irregular and approximates the shape of the tumor as closely as possible. The 12Gy yellow line is the treatment dose, also termed the “edge dose.” 12Gy of radiation is measured at the edge of the tumor. The green contours “5Gy” and “4Gy” reveal how the dose decreases as we move away from the edge. In this case, 4Gy is marked because it is considered the highest dose tolerated by the inner ear. If the tumor had grown laterally towards the inner ear, then the 4Gy line would be on the cochlea, marked with a red arrow. Hence, as in surgical treatments, the likelihood of hearing preservation is affected by tumor size and position. In surgery for larger tumors, hearing preservation is less likely due to microvascular compromise. In stereotactic radiation, it is due to the unwanted spread of the higher dose to the cochlea.

It is important to understand the radiation dose curves. If the tumor were a perfect sphere, it could be fully surrounded by the radiation sources, making the treatment plan simple. It would only require a single shot to cover the entire volume with radiation. A single shot has an optimum dose curve when the dose at the edge of the tumor is placed at exactly the 50% line. **Figure 3B** shows a dose curve for a single shot in a cross-sectional plane. Plans use 50% most often because this is where the spread of dose away from that point is minimized, corresponding to the steepest position on the curve. It also means that the maximal dose at the very center of the tumor (100%) is twice the dose at the edge (50%). This is desired, as the higher dose is limited to the center of the tumor, furthest away from surrounding structures. **Figure 3B** shows a theoretical y-axis dose curve from a single 4mm shot. With an edge dose of 12Gy conforming to the 50% dose line, 20Gy will fall at the 83.3% line. Why 20Gy? Because laboratory studies have been shown it to be the minimum lethal dose to human acoustic neuroma cells. If the green circle is the tumor edge, the red circle is the 20Gy line. Translating these circles into perfect spheres, the red center would contain 21.6% of the total green tumor volume. For this theoretical dose curve, 78.4% of the tumor volume would not receive a lethal dose. However, in practice, it is not so simple. The dose curves are not symmetric because the radiation cannot be delivered from below the head, as the body is in the way. Additionally, the source of radiation cannot be positioned below the tumor, as the head is connected to the body, and the body must enter the device from this angle. This skews the geometry of each shot. Furthermore, the tumor is not a perfect sphere. Multiple shots are given to conform the dose to the actual shape. When more than one shot is given, the phenomenon of “normalization” affects the dose curve by flattening the slope, effectively reducing the volume of the higher doses (48) (**Figure 3C**). **Figure 3C** demonstrates the effects of normalization clearly, showing that the center dose is dramatically reduced in volume when more than one shot is placed to cover the edge. Various methods exist to minimize the impact of normalization, but none can achieve a distribution similar to the ideal single shot.

Understanding radiation physics and biology is important because, over recent decades, the dose has been successively reduced to mitigate radiation side effects. Today, it is typical to give a 12Gy 50% edge dose. However, doses given in the 1990s and 2000s were much higher. In the 1990s, when Gamma Knife^®^ radiation became popular, a minimum 50% edge dose of 20Gy was used. This 20Gy dose is significant, as explained above, because it has been shown in laboratory studies that 20Gy is the minimum dose needed to cause cellular death in human schwannoma cells (49). Accordingly, patients were treated such that the entire tumor would receive at least this lethal dose. Unfortunately, they had poor outcomes with regard to radiation injury of the facial nerve, inner ear, and adjacent neural structures. These higher doses were initially deemed effective in controlling the tumor because the entire tumor was covered by a dose lethal to schwannoma cells. However, Nakamura et al. (2000) reported outcomes from the 1990s as follows: a dose of “20Gy achieved a good rate of tumor control with a low rate of cranial nerve dysfunction as compared to microsurgical resection at that time. However, the high radiation doses were associated with unacceptable complication rates relative to the improved results obtained by modern surgical techniques. Subsequently, radiation doses were significantly reduced [for emphasis] ***…***” (50). In the late 1990s, the first training programs in neurotology and cranial base surgery were accredited by the American Board of Medical Examiners. Accordingly, the microsurgical outcomes significantly improved with the broader use of transtemporal techniques. Good facial nerve outcomes are now expected in around 90% of patients with tumors of 3.0 cm and larger (28). Even better results with smaller tumors can be expected from experienced teams. The incidence of spinal fluid leak was reduced from 20% to 5% with transtemporal approaches (26, 27). At the same time, the hearing preservation techniques of MCF and retrolabyrinthine approaches were optimized and increasingly offered at more centers.

**Figure 4**.

**Figure 4:**
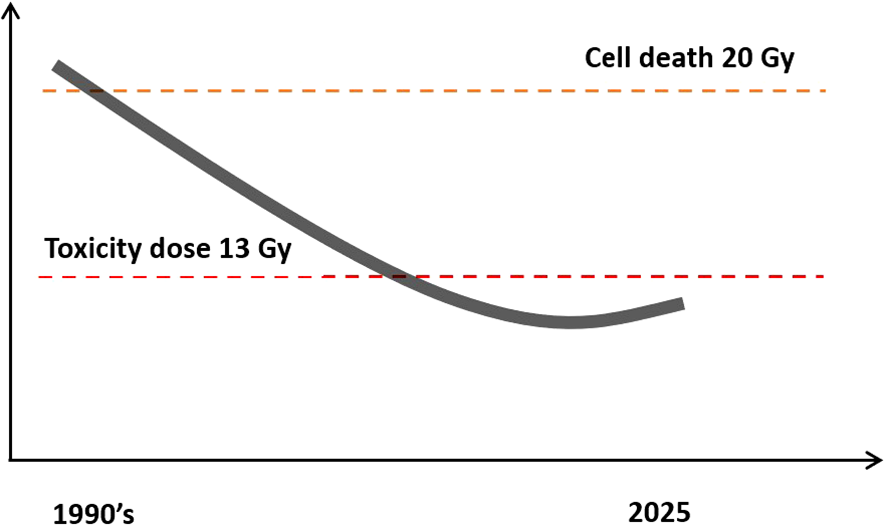
Trend of Gamma Knife^®^ treatment dose over time. Most centers today use doses under 13Gy because higher doses were found to be associated with excess radiation toxicity. The graph represents the trend of the treatment dose over time.

For the above reasons, the Leksell Gamma Knife^®^ was refined to provide better outcomes. To achieve this, centers worldwide moved towards lower dose levels. The dose in the late 1990s and 2000s reduced to 18, 16, 14, 13, and finally, as underscored by Vermeulen et al., who stated “from now, our center has established a protocol that limits the doses to 12Gy” (51). (**Figure 4**) Most centers worldwide now accept that the toxicity limit for Gamma Knife^®^ treatment of acoustic neuromas should be below 13Gy. Most neurotologists in the USA are certified in Gamma Knife^®^ and use it in appropriate patients.

The problem with the reduced dose is that most of the tumor volume is treated at a sublethal exposure, posing the real risk of future regrowth. It is common today to observe observe an initial swelling of the tumor. This can last up to a year and may require anti-inflammatory medications. This phenomenon can be particularly problematic in cystic tumors. Pendl et al. reported this in Stereotactic Functional Neurosurgery in 1996 and warned that 50% of patients with cystic acoustic neuromas required emergency surgery due to rapid enlargement and neurological compromise (52). These patients were treated with a mean dose of 13.8Gy, which is relatively low for the era. Once the swelling subsides, there is a period of involution, which is likely a consequence of the lethality of the dose at the very center. In essence, the radiation is thought to act as central debulking, accompanied by radiation-induced vasculitis to the remaining living cells (49). The question that remains is whether this effect endures over decades. To offer insight, Roberto Cueva from San Diego, California, USA, followed a series of over 100 patients treated with Gamma Knife^®^ at the lower doses of 12-13Gy over time. They concluded that there was no discernible significant difference between the growth patterns of untreated acoustic neuromas and those treated with stereotactic radiation at these reduced doses (53).

Experience performing microsurgery after Gamma Knife^®^ reveals that the lower the previous radiation dose, the more “normal” the surgical dissection will be. At higher doses, however, there is an increased chance of cranial nerve complications. Partial resections are more common after Gamma Knife^®^ due to adhesion of the facial nerve to the tumor. In a 2020 paper in the Journal of Neurosurgery, Mark Wiet at Rush University, Chicago, USA, reported a review of 300 patients who had undergone surgery for regrowth after Gamma Knife^®^. Only around half of the patients were able to receive a complete tumor resection due to this problem (54).

Finally, there is concern about the tumor transforming into malignancy. By 2014, nearly 30 such cases had been reported worldwide, though the real incidence is unknown. Patients with spontaneous malignant transformation and those initially presenting with atypical and malignant schwannomas without radiation were excluded (55). Seferis et al. reported that “radiation treatment increases the risk [of malignancy] by 10 times” and that the risk is “…even higher…” if the patient has NF2. NF2 is an autosomal dominant genetic illness, characterized by bilateral tumors and additional meningiomas in the surrounding brain and spine (56). Evans et al. reported on over 250 patients treated with radiation for NF2. The 20-year malignancy rate was 6% They concluded that NF2 patients should **not** be offered radiotherapy as first-line treatment for their tumors (57).

There is also the further risk of radiation-induced meningioma (RIM), which can later develop into higher grades of biological aggressiveness. We have treated a number of these unfortunate cases. They typically appear in the NF2 population that was radiated as children with the Gamma Knife^®^. Unfortunately, the necessary microsurgical removal of these tumors is complicated by infiltration of adjacent nerves with these aggressive tumors. These highly complicated cases are likely under-represented in the literature as they often meet exclusion criteria in broader studies.

### 5.3 Microsurgical case studies

#### 5.3.1 Case example of middle fossa hearing preservation surgery

A 40-year-old female initially presented with ***left*** facial pain in a pattern classic for trigeminal neuralgia. She was referred by the neurology department because medical therapy was not controlling the symptoms. On further questioning, she admitted to a few prior episodes of vertigo that spontaneously resolved. Careful review of the MRI revealed a small 3 mm tumor on the contralateral right side of the vestibular nerve, consistent with a small acoustic neuroma. (**Figure 2A**). The patient had normal hearing levels, and the vertigo had resolved for the present. As her primary complaint was the facial pain, this was addressed successfully with a microvascular decompression by our team using a minimally invasive keyhole approach. At the planned follow-up, we further addressed the treatment options for the small acoustic neuroma. Continued observation with serial imaging was offered. Gamma Knife^®^ was discussed, and the expected outcomes were reviewed carefully. Finally, the option of middle cranial fossa (MCF) microsurgery was presented along with the appropriate risk profile.

The patient was young, and the tumor, though small, was located close to the cochlea. This proximity makes the option of radiation less appealing, given the association with poor long-term hearing. The decision-making at this point demanded a comparison of the surgical risks within a young healthy patient against the risks and outcomes of observation over time. While it is admittedly impossible to know exactly what the growth pattern of this tumor would have been, it was safe to assume it would grow over years or decades, with hearing expected to decline as the tumor enlarged. The risks of middle cranial fossa surgery in this patient without comorbidities carry a less than 1% risk of major events, an excellent chance for a normal facial nerve outcome, and a very good chance of normal hearing.

For patients with very small acoustic neuromas, the likelihood of hearing preservation from surgery is very high and safely estimated at around 80% in our experience. Tumors at this small size have yet to form significant adhesions to the surrounding nerves and blood vessels. The smallest of tumors presents a unique window of opportunity for the easiest removal. This factor cannot easily be extracted from the literature, as tumor size is often grouped as 10mm or less.

Ultimately, the patient chose to undergo surgery. She had a total resection with excellent hearing and completely normal facial function. She had early cerebrospinal fluid (CSF) leakage from the right nostril on post-operative day 2. A lumbar drain was placed at the bedside and remained for 5 days, resolving the issue without incident. Resolution of CSF leak after lumbar diversion is expected in 87% (26) of cases. In our report published in Laryngoscope, none of the patients treated with lumbar diversion had meningitis, nor did the 13% who underwent fat replacement.

One major under-reported benefit of microsurgery is the psychological relief of knowing that the tumor has been removed and no longer poses a risk. This major concern for patients, which cannot easily be quantified in clinical reports, can only be addressed through surgery.

### 5.4 Decision making for a 79 y/o patient with dizziness

A 79-year-old female was referred after undergoing 6 months of vestibular physical therapy for worsening imbalance. The therapy was ineffective. Despite her advanced age, she was in good medical condition. She was previously active as a farmer and lived alone. She now found herself homebound and afraid to leave home and drive a motor vehicle because of fear of falling. There was a 1.2 cm left-sided acoustic neuroma on MRI. She had left-sided high-frequency sensory neural hearing loss with a deficit in speech understanding out of proportion to the pure tone abilities. She reported a gradual change in hearing in the left ear with distortion. She was recommended a hearing aid but found it ineffective. Her pure tone average was normal, but her speech understanding dropped to 64% in less than a year. Rotational chair testing revealed a significant reduction in vestibulo-ocular reflex (VOR) gain and a 65% left-sided caloric weakness, indicating an uncompensated left-sided peripheral vestibular lesion. The patient had seen several doctors in the past year and had been told she was too old to safely recover from surgery. She was referred to us by her physical therapist for a second opinion.

Though it is generally accepted that age affects the speed and extent of vestibular compensation, her otherwise healthy state suggested that treatment with surgery should be considered, given the severity of her imbalance. She is right-handed and has a left-sided tumor. Though she has Gardner–Robertson class two serviceable hearing, there are a few relative contraindications to MCF hearing preservation surgery. First, the primary objective of this surgery is to treat her life-altering vestibular disease, for which the best treatment is translabyrinthine surgery. With this method, the entire vestibular apparatus is ablated, and the vestibular nerve is completely sectioned. With any hearing preservation method, there is a tendency to retain attachments, making vestibular complaints afterwards more likely as the tumors are smaller and the balance organ is left in place. These symptoms generally resolve following vestibular rehabilitation. For patients presenting with primary uncompensated vestibulopathy, this could be a very undesirable outcome. Furthermore, in the older age group, elevation of the left dominant temporal lobe may be associated with excessive swelling and post-compressive symptoms.

Gamma Knife^®^ treatment was discussed and deemed to be unwise, given the primary complaint of disabling vestibulopathy. Gamma Knife^®^ would NOT induce a complete vestibular lesion, which permits a better functional outcome with vestibular adaptation, compensation, and substitution mechanisms induced by a complete unilateral loss. Therefore, Gamma Knife^®^ radiation, though easily offered and executed, could exacerbate the problem by inflaming the nerve with radiation-induced vascular injury.

Ultimately, the patient underwent left-sided translabyrinthine surgery with complete removal of the acoustic neuroma, along with a labyrinthectomy and complete sectioning of the cochleovestibular nerve. The patient had completely normal facial function after surgery and no complications of spinal fluid leak. One month after surgery, she was confident driving and reported complete resolution of her imbalance.

### 5.5 Case example of retrolabyrinthine pre-sigmoid hearing preservation surgery

A 29-year-old female presented with left SSNHL, which occurred three months prior. She was treated with corticosteroid therapy, both parenteral and intratympanic, and recovered from moderate to mild sensory neural hearing loss. She had a pure tone average of 30 decibels and 100% speech discrimination. Her MRI showed a 1.3-centimeter left-sided cerebellopontine angle (CPA) mass consistent with acoustic neuroma.

**Figure 5**.

**Figure 5:**
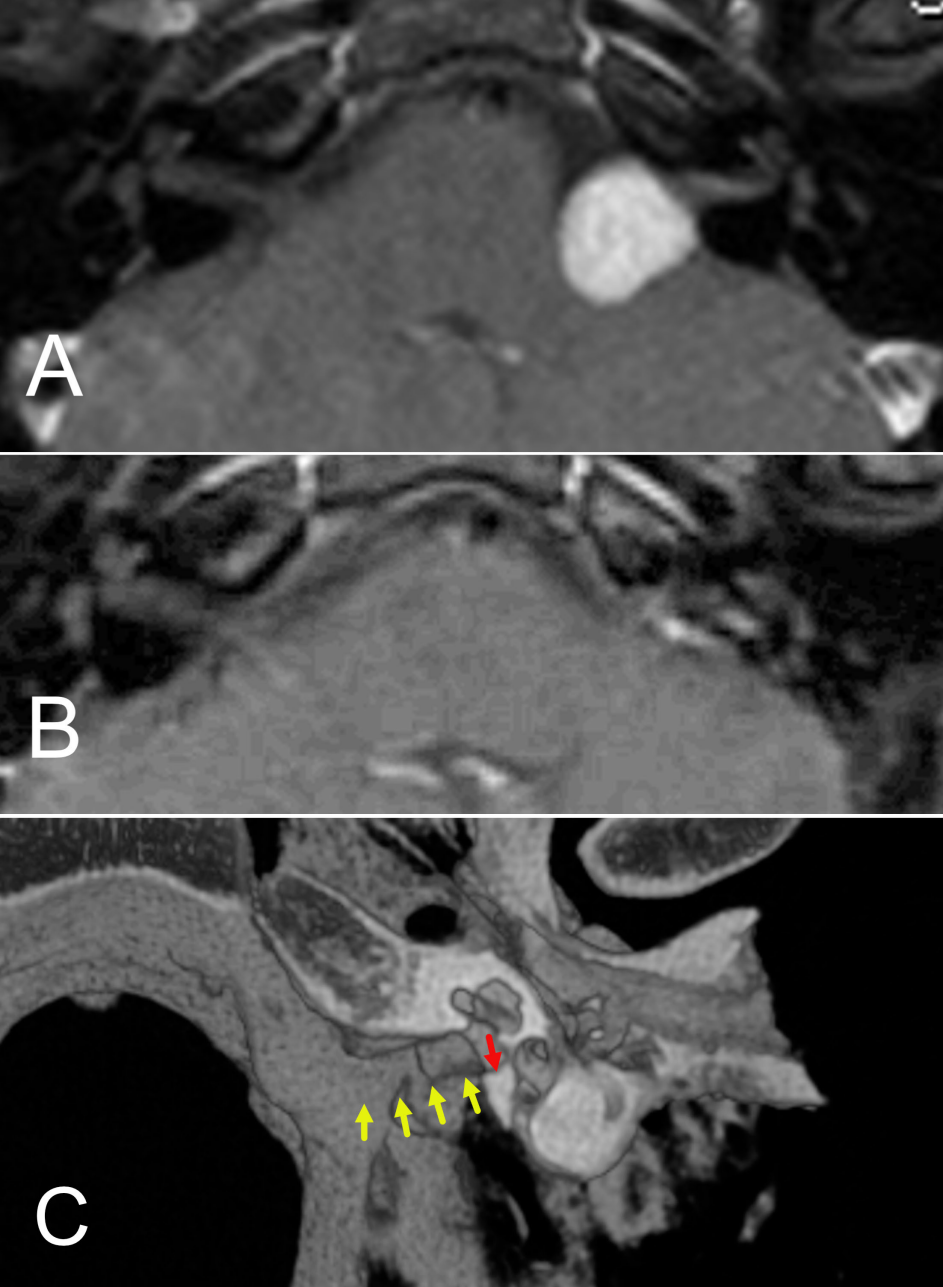
**(A, B)** Preoperative **(A)** and postoperative **(B)** MRI of a patient who underwent retrolabyrinthine hearing preservation of an acoustic neuroma. **(C)** 3D reconstruction of post operative CT scan for the patient operated by the retrolabyrinthine approach. The yellow arrows show the degree of internal auditory canal bone removal. The red arrow shows the final portion of bone that remained. At least 4/5 of the length of the internal canal can be removed and the inner ear structures retained.

The tumor was positioned mostly medially, with limited volume inside the internal auditory canal. The mass mildly indented the brainstem. Though the size of the tumor was measured as 1.3 centimeters in the largest diameter across, the intracranial component behaved more like a 2.3-centimeter tumor. Tumors are typically measured, including the full extent of the internal auditory canal and then traced medially towards the brainstem. This underscores the importance of noting both the position and the longest diameter when describing tumor size. The patient’s case was reviewed by our radiation oncologists, who expressed hesitancy to offer the patient radiation because of her young age and the tumor’s brainstem contact. It was, therefore, decided that surgery was indicated. A retrolabyrinthine approach for tumor removal and hearing preservation was recommended. This approach is a variation of the translabyrinthine technique but retains the balance canals while still exposing the medial 3/4 to 4/5 of the internal auditory canal (58). (**Figure 5C**). The surgery was uneventful, resulting in total tumor removal. (**Figure 5B**). Though there was a reduction of the pure tone average to 45 decibels, speech discrimination remained normal at 92%. The patient had excellent hearing with a hearing aid. Facial nerve function was fully retained.

### 5.6 Expanded transtemporal approach for large CPA tumors

The translabyrinthine approach is our preferred surgery when hearing preservation is no longer an objective. It offers safe and wide access for tumors of all sizes. However, some patients benefit from combining the translabyrinthine approach with a subtotal petrosectomy and closure of the external auditory canal. This gives an even wider view, which is desired for the largest of tumors. Removal of the cochlea can be added, giving better access to the anterior part of the tumor.

**Figure 6**.

**Figure 6:**
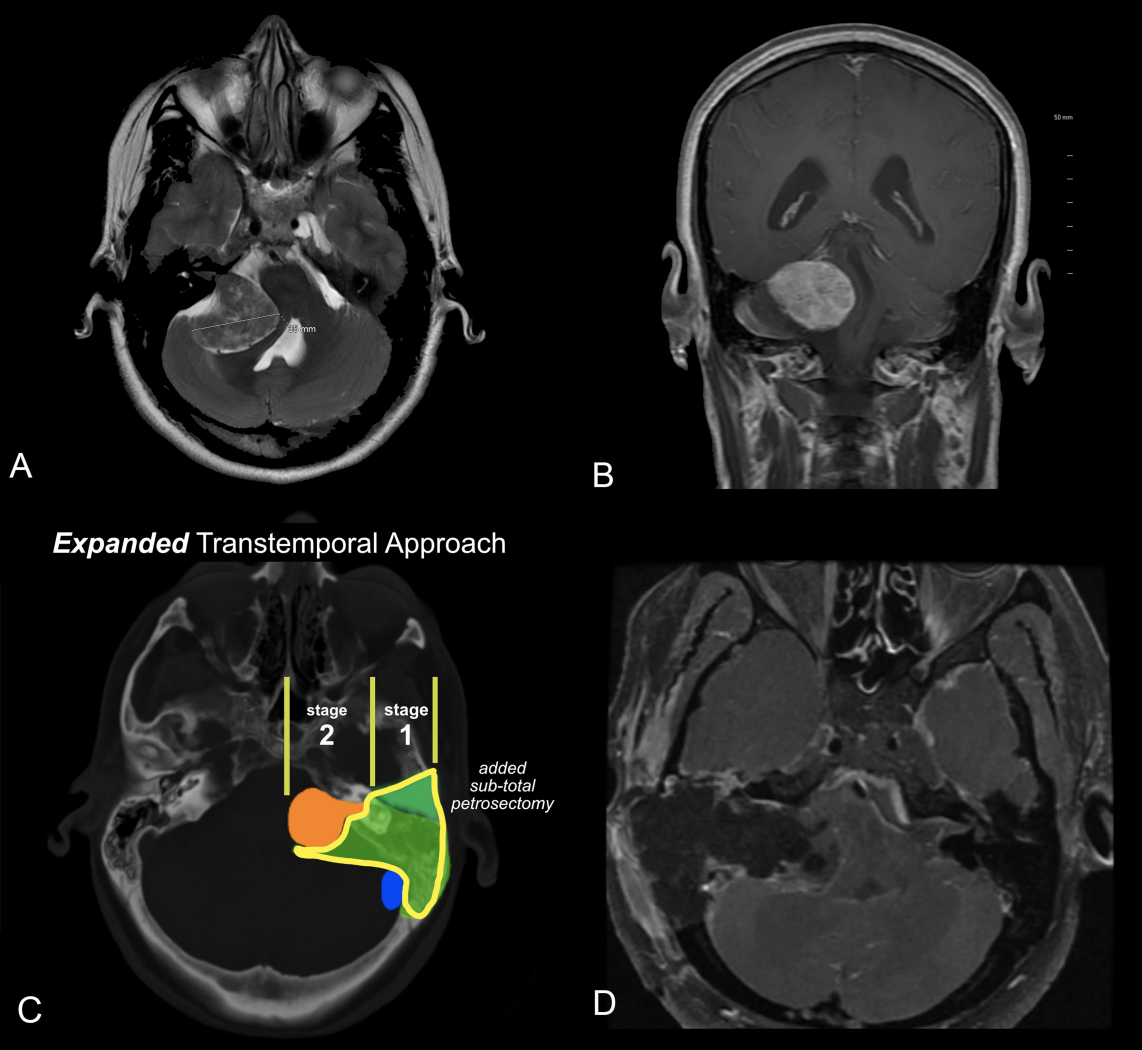
**(A, B)** MRI of a patient with a very large acoustic neuroma. **(A)** T2 axial MRI. **(B)** T1 C+ coronal MRI. **(C)** Expanded Transtemporal Approach. The green segment, lower portion, is the same as the translabyrinthine resection. The upper green segment is the additional bone removed from the subtotal petrosectomy. The lateral bone removal can easily be performed as a separate stage from the tumor removal to optimize safety and outcomes. **(D)** Post-operative MRI revealing gross total removal by the expanded transtemporal approach.

In most cases, the petrosectomy and translabyrinthine dissection can be performed as a first stage, and the definitive removal of the tumor performed as a second stage. (**Figure 6C**). This approach allows the patient to recover from imbalance and vertigo, and to more quickly ambulate after the subsequent tumor removal. Additionally, the risk of the CSF leak is minimized as the two main routes of leakage, the eustachian tube and external ear, are closed and fully scarified before the CSF is exposed in the second stage.

A 38-year-old female presented to the emergency department with headache and worsening gait disturbance over the past month. She reported a long history of right-sided hearing loss. An MRI revealed a very large tumor greater than 4 cm in maximal diameter, with an MRI appearance consistent with acoustic neuroma. (**Figure 6 A, B**).

Given the size of the tumor, a decision was made to offer the patient an expanded transtemporal approach, followed by resection the following week. The patient required continued hospitalization as a precaution, given the extent of brainstem compression. After the first stage of surgery, the patient experiences only minor vestibulopathy. This is often seen with larger tumors that have already destroyed the vestibular function of the affected side. After the first stage, there was a partial improvement in gait because the bone removal allowed some decompression of the brainstem. The patient underwent a second-stage total tumor removal. She had partial facial nerve paresis that recovered to normal over the next 6 months. There was complete resolution of the gait disturbance and no complications.

## 6 Summary

Acoustic neuromas are likely more common than previously recognized. High suspicion is warranted in patients with subtle complaints. Early diagnosis, along with advancements in microsurgical techniques, affords patients more options and the potential for durable hearing preservation. Treatment planning must be considered on an individual basis.
